# Technologies for Mechanical Recycling of Carbon Fiber-Reinforced Polymers (CFRP) Composites: End Mill, High-Energy Ball Milling, and Ultrasonication

**DOI:** 10.3390/polym16162350

**Published:** 2024-08-20

**Authors:** Enrique Martínez-Franco, Victor Alfonzo Gomez Culebro, E. A. Franco-Urquiza

**Affiliations:** 1 Manufacturing Department, Center for Engineering and Industrial Development, Av. Pie de la Cuesta 702-No. 702, Desarrollo San Pablo, Santiago de Querétaro 76125, Mexico; enrique.martinez@cidesi.edu.mx; 2 Aerospace Section, Center for Engineering and Industrial Development, Carretera Estatal 200, km 23, Queretaro 76270, Mexico; victor.gomez@cidesi.edu.mx

**Keywords:** mechanical recycling, carbon fiber-reinforced polymers, end milling, high-energy ball milling, ultrasonication

## Abstract

Carbon fiber reinforced polymer (CFRP) composites have very high specific properties, which is why they are used in the aerospace, wind power, and sports sectors. However, the high consumption of CFRP compounds leads to a high volume of waste, and it is necessary to formulate mechanical recycling strategies for these materials at the end of their useful life. The recycling differences between cutting-end mills and high-energy ball milling (HEBM) were evaluated. HEBM recycling allowed us to obtain small recycled particles, but separating their components, carbon fiber, epoxy resin, and CFRP particles, was impossible. In the case of mill recycling, these were obtained directly from cutting a CFRP composite laminate. The recycled materials resulted in a combination of long fibers and micrometric particles—a sieving step allowed for more homogeneous residues. Although long, individual carbon fibers can pass through the sieve. Ultrasonication did not significantly affect HEBM recyclates because of the high energy they are subjected to during the grinding process, but it was influential on end mill recyclates. The ultrasonication amplitude notably impacted the separation of the epoxy resin from the carbon fiber. The end mill and HEBM waste production process promote the presence of trapped air and electrostatics, which allows recyclates to float in water and be hydrophobic.

## 1. Introduction

Carbon fiber composite materials are manufactured by impregnating carbon fiber fabrics with epoxy resins to obtain carbon fiber-reinforced polymer (CFRP) composites. CFRPs have very high specific properties, so they play an essential role in the growth of various high-tech sectors such as aerospace, wind, and sports. However, the high consumption of CFRP leads to a high volume of waste, so it is necessary to formulate viable recycling strategies for these technological materials at the end of their useful life [1]. Academia and industry combine efforts to improve the efficiency of resource consumption for global sustainable development and environmental conservation, aligned with the circular economy [2,3,4,5]. An example of the above is the European Community, which aims at the recovery, reuse, and recycling of almost all components in vehicles at the end of their life cycle [6,7,8], and similar decisions have been made in the aerospace industry [9]. 

CFRP currently constitutes more than 50% of the structural mass of commercial aircraft and will reach the end of life (EoL) in 20–30 years [10]. Therefore, the question arises about how these technological materials will be recycled.

The main drawback of CFRP is that thermosetting resins form cross-linked structures during their curing process, so they cannot be melted and reprocessed [11]. Additionally, carbon fibers are abrasive. The management of CFRP waste is mainly carried out through disposal and recycling. Disposal is the most common method because waste is taken to landfills [12,13]. Thermal recycling, which involves energy recovery, commonly known as pyrolysis, consists of incinerating waste to take advantage of the gases produced by combustion [14,15]. Chemical recycling, known as solvolysis, uses chemical solutions to remove the epoxy resin from CFRP. This process is relatively cheaper than heartburn, although it is more polluting and risky since it uses typically dangerous chemicals. In both recycling processes, the thermosetting resin (which has a high thermomechanical potential) is eliminated, and only the carbon fiber is recovered, which is damaged by the harsh processes that considerably reduce its mechanical properties.

Mechanical recycling recovers both the thermosetting resin and the carbon fiber without damaging them, as it involves a primary low-speed cutting and grinding process to reduce the size of the CFRP into pieces in the range of 50 to 100 mm [16,17]. Subsequently, these crushed pieces are ground to obtain particles with a size distribution of 10 to 1 mm [18,19]. M. Durante and co-workers [1] reported that mechanical recycling represents the most promising way to recover carbon fibers from the polymeric matrix of the CFRP components since this methodology does not require burning at high temperatures or chemical substances to decompose the polymeric matrix. 

Mechanical recycling allows the recovery of three types of waste: short carbon fiber, epoxy resin powder, and CFRP microparticles [18]. Mechanical recycling of CFRP does not induce structural damage to the carbon fiber and provides high expectations of reuse and sustainable savings to develop new materials or products, taking advantage of the intrinsic properties of carbon fiber and epoxy resin.

The most used and well-known mechanical recycling technology is the one that uses end mill tools coated with carbides (carbide end mills) because of the high abrasion of CFRP. This technology is relatively the most accessible, requiring only a conventional small helix end mill. The tool follows a programmed trajectory using computer-aided manufacturing systems. Nonetheless, the main restriction is tool wear and difficulty milling curved parts. Norbert Geier and collaborators [19] conducted a complete review of edge-cutting technologies in CFRP composites. The review article is oriented toward cutting efficiency in composites and is helpful in guiding efforts toward mechanical recycling of CFRP. Other research explored various configurations of milling tools to increase end mill tool life [17,20]. Process conditions such as orientation, speed, and cutting feed have also been evaluated, with feed speed having the most significant effect, followed by cutting speed, on tool wear [18,21]. Hosokawa et al. [22] examined the effect of tool coatings. The force analysis showed that the tangential and normal force components decrease with increasing helix angle.

Mechanical milling is a technique used to convert long carbon fibers into short fibers [23]. Ball and hammer mills are the most common methods for converting dry carbon fiber into powder. In the ball milling process, carbon fiber is placed in a drum that houses high-hardness (usually stainless steel) and high-density spheres. Through the rotation of the drum, the balls begin to move in a cascade fashion and collide with the surrounding material, causing localized high-pressure impacts that grind the fibers into powder.

High energy ball milling (HEBM) is a complex process that involves a high speed of ball movement, also causing collisions and friction between them that favor homogeneous mixing, promote morphology changes, and influence the evolution of defects of the crystal lattice and formation of new phases. Thus, HEBM mainly synthesizes powder products to develop materials and coatings [24]. Although HEBM has been employed to fabricate ultrafine graphite waste [25], the technique has not been explored for recycling CFRP composites.

Another technique that can be used to provide an additional step in the mechanical recycling of CFRP composites is ultrasound. Ultrasonic treatment can alter the surface roughness characteristics of particles and promote their efficient separation by flotation [26].

On the other hand, the sonication process uses ultrasonic sound waves that produce thousands of microscopic vacuum bubbles in the solution because of the applied pressure. The bubbles collapse in solution during the cavitation process, leading to the generation of enormous energy. This way, ultrasonic cavitation leads to solid particles’ dispersion, homogenization, and disintegration. Ultrasonication has been used to disperse nanoparticles in a polymer matrix [27]. The platelets that make up the mineral clays [28,29] can be exfoliated using the ultrasonication technique to increase the thermal and mechanical properties of the polymers [30,31]. Ultrasonication is also used to disperse the nanoclay platelets that reinforce CFRP composites by adding small percentages by weight (<5 wt %) to the epoxy resin [27]. Other nanoparticles have reinforced fiber-reinforced polymer composites [32]. Hiroaki Miyagawa et al. [33] investigated the mechanical and thermophysical properties of biobased epoxy nanocomposites reinforced with organo-montmorillonite clay and carbon fibers. They used sonication to process the organically modified clay into bio-based glassy epoxy networks, which resulted in the clay nanoplatelets being homogeneously dispersed and thoroughly exfoliated in the matrix. There is no evidence of ultrasonication reducing the particle size of recycled CFRP (rCFRP) composites at the moment. 

This work uses different technologies to obtain recycled materials from CFRP composites. The first is the most widely used to cut this composite type using end milling cutters. End milling is used more to finish composites than to destroy them. This is because the cutter edge wears down quickly. However, using end mills to evaluate the mechanical recycling capacity is convenient. The recycled materials obtained comprise short carbon fiber, epoxy resin powder, and rCFRP particles. The second technique is unconventional since the HEBM technique is used in this work to pulverize the recyclates. This technique is complementary to assessing the efficiency of obtaining finer and smaller recycled particles. The recycled materials obtained by end milling and grinding are subjected to ultrasonication to separate epoxy resin residues from the carbon fibers. The morphology of microscopic CFRP particles was inspected after cutting processes with end mills and high-energy ball milling. Recycled materials could be used as efficient reinforcements since mechanical recycling does not alter the structural capacity of CFRP composites [18], opening up a range of possibilities for research and development of new products, including large-scale recycling technology.

## 2. Materials and Methods

The CFRP composites consisted of a 1 × 1 3K taffeta carbon fiber fabric weighing 198 g/m^2^, supplied by Quintum. The carbon fabric has a tensile strength of 2137 MPa and an elastic modulus of 227 GPa. The epoxy resin Epolam 2015 from Sika was used as the polymer matrix. According to the manufacturer’s technical sheet, Epolam 2015 has a viscosity (25 °C) of 1680 mPa-s and a density of 1.15 g/cc. The hardener Epolam 2015, with amine groups, was used as a catalyst in a mixing ratio of 32 by weight with a useful life of 140 min at room temperature (25 °C).

Carbon fiber and epoxy resin laminates were manufactured in a controlled environment using the vacuum-assisted resin infusion (VARI) method. The negative pressure was 2670 Pa, and the resin curing reaction took approximately 24 h (~25 °C). The orientation of the carbon fiber fabrics was quasi-isotropic, with a stacking sequence of [{0/90/ ± 45/0/90/ ± 45}_2_/{0/90}]_s_. The CFRP laminates had 18 layers of carbon fiber plain weave fabric and nominal dimensions of 300 mm × 300 mm × 4 mm. More information on manufacturing CFRP laminates can be found in previous work [18].

For mechanical recycling technology using end mill cutters, CFRP laminates of 90 mm × 250 mm were placed horizontally on a Challenger CNC model MM-430. Carbide end mill cutters of 10 mm diameter and four flutes were used to cut the lateral part of the laminate. Each section had one cutter, and the laminates were firmly clamped to prevent vibrations or slipping during cutting. In a previous work [18], the amount of mechanically recycled CFRP (rCFRP) collected was measured as a function of the cutting speed, ranging from 1100 rpm, 1800 rpm, and 2500 rpm. The rCFRP was separated on a Ro-Tap laboratory sieve equipped with W.S. sieves to obtain the recyclates composed of short carbon fiber, epoxy resin powder, and rCFRP microparticles. 

The recyclates were ground using HEBM technology. For this, a Simoloyer CM01-2l horizontal milling was used. Samples were taken at different grinding times as in previous experimental tests. The final processing time was determined to be 40 min.

In order to separate the recyclates, two experimental tests were carried out by taking samples of 1 and 2 g of recyclates in a beaker containing 500 mL of tap water. Samples were placed on a hot plate magnetic stirrer (Thermo Fisher Scientific, Waltham, MA, USA) and subjected to 350 rpm and 510 rpm, respectively. The tests were carried out at room temperature for 40 min. Subsequently, the samples were placed inside an Autoscience AS2060B (Pontiac, MI, USA) ultrasonic bath at 47 kHz for 40 min.

The ultrasonication technique was used using a QSONICA Q700 (Newton, CT, USA) ultrasonicator to evaluate the separation capacity of the rCFRP particles. The test involved placing 1 g and 2 g of rCFRP in 200 mL of tap water inside a beaker. A 24.5 mm diameter probe was used, and tests were performed varying the amplitude of 50 and 75% for 30 min to avoid excessive water heating.

The morphology of the rCFRP processed by the various mechanical recycling technologies such as end mill, HEBM, and ultrasonication was observed in a JEOL 6610LV scanning electron microscope (SEM) at 10 kV. For the SEM characterization, powders were (Dearborn Road Peabody, MA, USA) metalized using a JEOL Smart coater DII-29010SCTR that is operated under vacuum from a mechanical pump and provides a gold coating. This equipment generates a cloud ion from a current source of 5 A and a power of 500 VA. The parameter in the equipment is the sputtering time, which was adequate for the best observation of powders under SEM, and it was 5 min. 

The chemical composition of the rCFRPs was determined by X-ray fluorescence (XRF) on a Spectro Xepos (Kleve, Germany). Five different samples were measured, and the average value obtained was presented.

## 3. Results and Discussions

**Figure 1:**
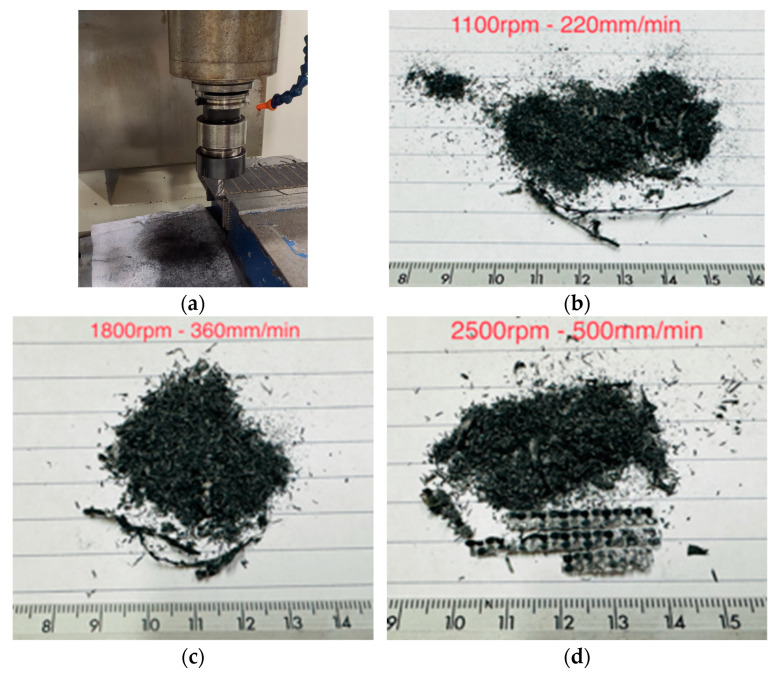
Photographs of the end milling recyclates obtained at different operation conditions: (**a**) edge milling, (**b**) 1100 rpm, (**c**) 1800 rpm, and (**d**) 2500 rpm.

Figure 1 shows the rCFRP obtained after the end milling process applying different velocities of the tool. The cut was made in the lateral zone of the CFRP laminates (Figure 1a) [18]. It is relevant to highlight that only the recyclates deposited on the white sheet of paper were collected and analyzed. Particles ejected into the air or deposited outside the leaf were not analyzed. Furthermore, it is highly recommended that cutting and grinding of the compounds be carried out in a closed room to avoid the volatility of micro and nanometric-sized recyclates that could cause damage to the health of the workers. Particle detectors in the air are also recommended to check the air quality of the cutting area and detect the concentration of particles to which the workers would be subjected.

The end mill recyclates obtained are small particles of different sizes. However, cutting speeds seem to influence end mill recyclates. For example, cutting speeds of 1100 and 1800 rpm promote chip detachment or fiber pullout (Figure 1b,c), while at 2500 rpm, small pieces of sheets are obtained, as seen in Figure 1d.

The presence of waste in the form of fiber pullout or delamination during the milling of CFRP composites has been reported by other authors and is attributable to defects induced by the cutting process [34]. It is relevant to highlight that most of the research evaluates the effect of cutting on the machining quality of CFRP composites and not on mechanical recycling [21,35]. In a previous work [18], the presence of fiber pullout and delaminations indicate the beginning of tool wear. However, delamination also seems to occur due to the effect of cutting speed. In their research, Pascual et al. [36] concluded that the axial cutting force is mainly responsible for the formation of delamination by debonding and expulsion induced by machining. Therefore, the evaluation of recycling technologies in this work will be carried out using only the recycling obtained at 1100 rpm.

**Figure 2:**
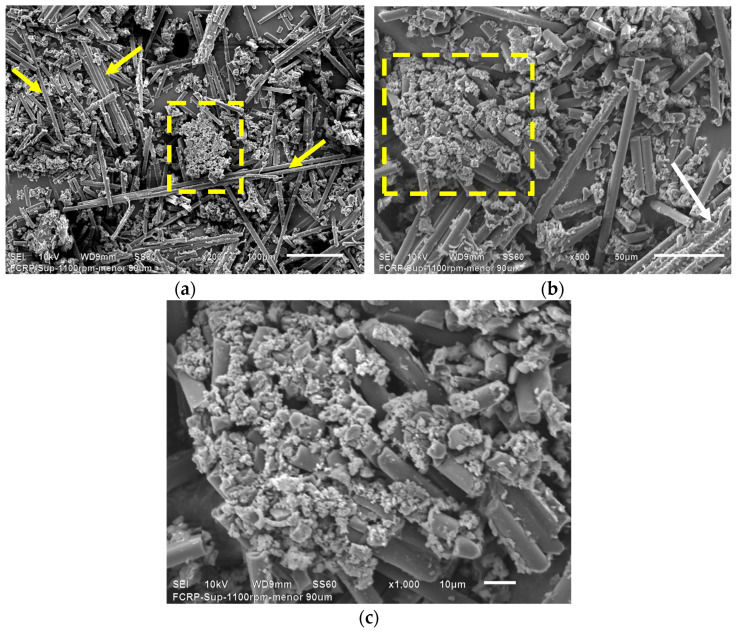
SEM micrographs corresponding to the sieved end mill recyclates. Features of recyclates can be observed at different magnifications: (**a**) 200×, (**b**) 500×, (**c**) 1000×. The yellow arrows indicate long fibers, the dashed box highlights agglomerated particles, and the white arrow indicates rCFRP particles.

The end mill recyclates at 1100 rpm were sieved using two meshes of 90–425 microns. Figure 2 presents the SEM micrographs corresponding to the end mill recyclates. 

It is possible to see long carbon fibers indicated by yellow arrows, which managed to pass through the 90 and 425-micron mesh during the sieving process. Agglomerated particles framed in the yellow dashed box are also seen (Figure 2a). At higher magnifications (Figure 2b), rCFRP particles indicated by a withe arrow can be seen. The rCFRP particles are made up of aligned carbon fibers joined together with epoxy resin. Meanwhile, the agglomerates are composed of shorter carbon fiber with a large amount of epoxy resin, as observed in Figure 2c. Figure 2c shows the characteristic features of the agglomerated particles, where epoxy resin particle residues agglomerate a considerable amount of short carbon fibers with a heterogeneous distribution.

**Figure 3:**
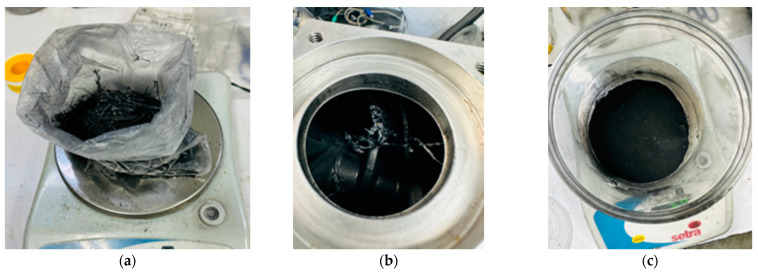
Photographs corresponding to the HEBM processing: (**a**) material weighing, (**b**) before the start of HEBM, (**c**) after the HEBM procedure.

Figure 3 presents the HEBM procedure performed. The end mill recyclates (1100 rpm, without sieving, and containing large fibers pullout) were milled using HEBM technology at a rotor speed of 860 rpm and a processing time of 40 min.

**Figure 4:**
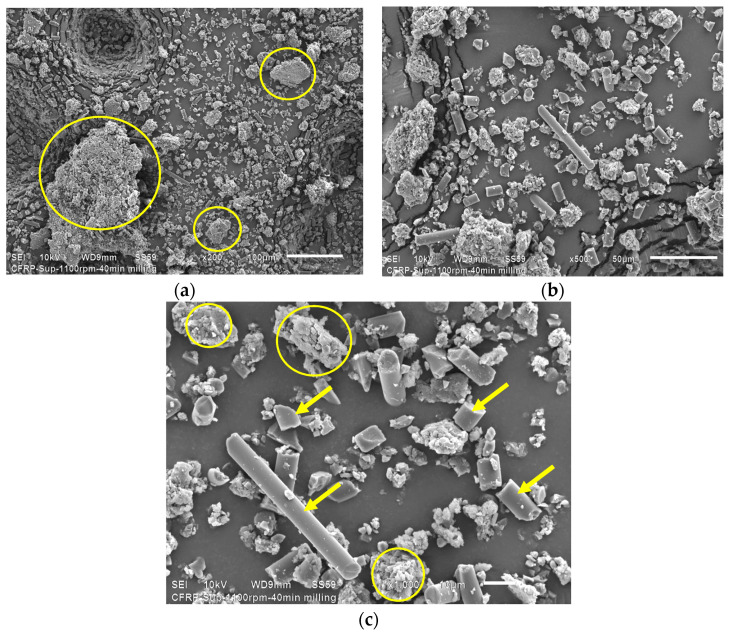
SEM micrographs corresponding to the HEBM recyclates. Features of recyclates can be observed at different magnifications: (**a**) 200×, (**b**) 500×, (**c**) 1000×. The yellow circles highlight agglomerated particles, and the yellow arrows indicate single carbon fibers and graphite particles.

The as-is recyclate obtained from side milling of the CFRP laminates was weighed on a laboratory scale (Figure 3a), then the recyclate was placed inside the mill, and the HEBM process was started (Figure 3b). After milling, the recyclate powder was extracted, as shown in Figure 3c. Figure 4 presents the SEM micrographs that reveal the morphology of the HEBM recyclates.

From HEBM recycling, it is possible to observe three types of waste: single short carbon fibers or graphite particles, and epoxy particles. The latter can be seen at low magnifications, indicated with yellow circles in Figure 4c. The HEBM process produces more agglomerated epoxy resin particles than the end milling process, where the agglomerates are micrometric resin particles containing randomly distributed short carbon fibers.

**Figure 5:**
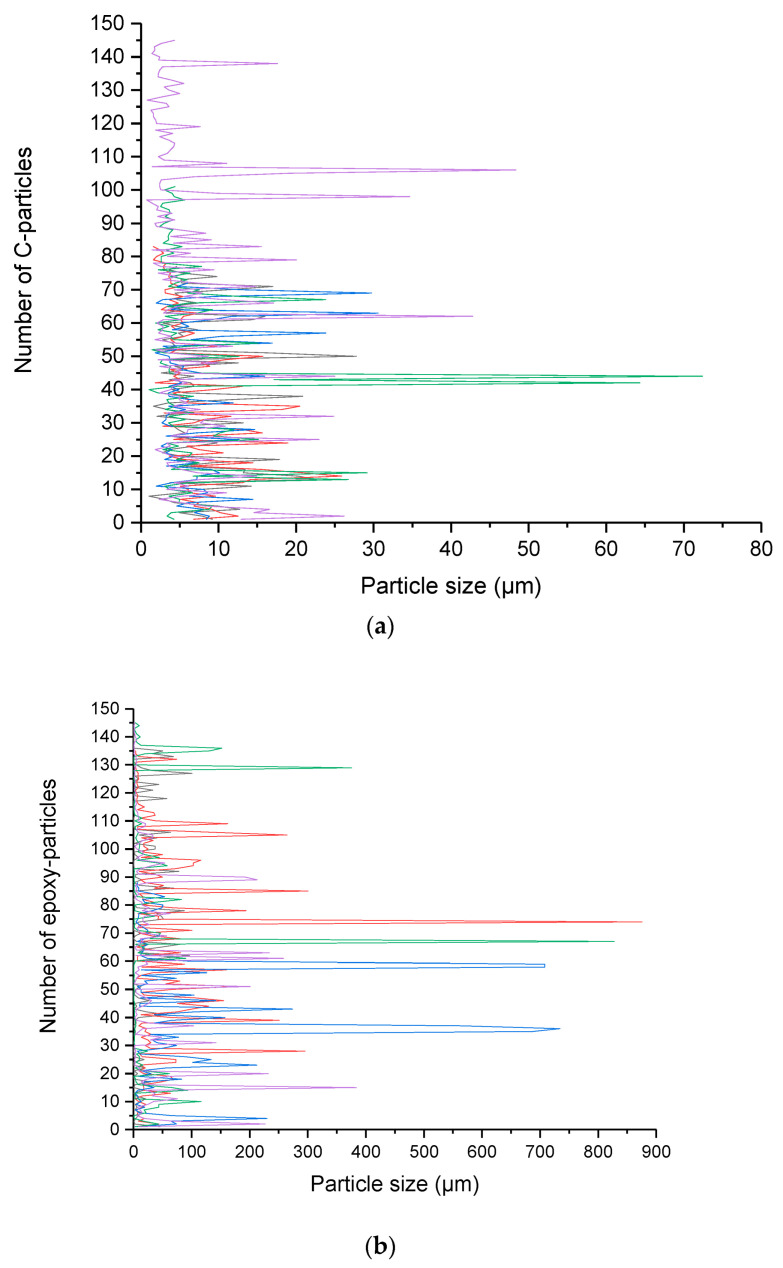
Particle size distribution of powder obtained after HEBM by using ImageJ free software, (**a**) C-particles and (**b**) epoxy particles. Colors are related to different SEM images.

At high magnifications (Figure 4b), individual long carbon fibers, graphite particles, and agglomerated epoxy particles are visible. After the HEBM process, the carbon fibers’ length is dramatically reduced compared with recycled end mills, obtaining graphite powder of fewer than 10 microns. Figure 4c shows that the short carbon fibers and the graphite particles contain traces of epoxy resin on their surface, which confirms the high adhesion between both constituents produced by the molecular compatibility between the carbon fiber and the epoxy matrix in the CFRP compounds. Particle size evaluation of powder obtained by HEBM was performed using the ImageJ free software (https://ij.imjoy.io/ access on 8 July 2024) using five SEM images, and two types of particles were considered: C-fiber and epoxy. Results are shown in Figure 5.

Figure 5 shows a significant difference in particle size compared with C-fibers, some up to 80 microns, vs. epoxy, which has particles near 900 microns. However, the average size of particles is 7.06 and 32.86 microns of C-fibers and epoxy, respectively. The average of C-fibers is considered to be in the range of submicrometric size. 

On the other hand, mechanical recycling of CFRP compounds entails contamination promoted by the wear of cutting and grinding tools. The elements mostly present in tools are chromium (Cr), iron (Fe), and cobalt (Co). The XRF technique was used to detect these elements. Table 1 presents the chemical composition of the recyclates obtained through end milling and the HEBM process.

As expected, the most significant elemental contaminants come from iron, which is the base manufacturing material for cutting tools and grinding balls. Curiously, the end milling-cutting process does not entail excessive contamination. In contrast, the HEBM process produces considerable elemental contaminants, even more so if mechanical recycling is considered to be evaluated at the laboratory level (that is, grams of material). Therefore, it is essential to emphasize that the presence of these elements, mainly Fe, should not be ignored when using recyclates to manufacture new materials through melt welding processes since it could alter the properties of the materials.

**Figure 6:**
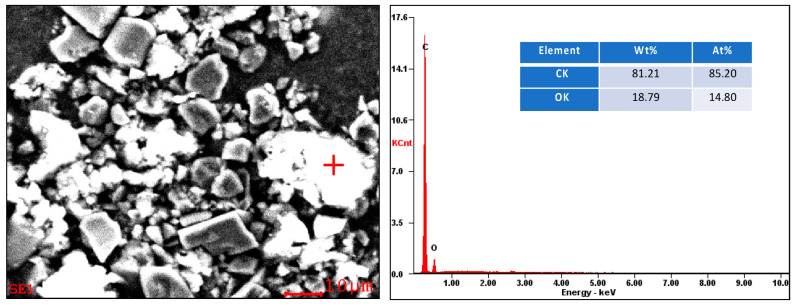
SEM-EDS punctual chemical analysis showing the presence of epoxy resin in milled powder.

Specific punctual chemical analysis using an Energy Dispersive Spectrometer (EDS) of SEM equipment was performed directly on the powder obtained after HEBM. The evaluation was performed to confirm epoxy resin particles as part of the product. Additional/complimentary results are located in the Supporting Information section. Then, Figure 6 shows the punctual EDS evaluation in an epoxy particle. 

SEM-EDS chemical analysis results confirm the presence of epoxy particles separated from carbon fibers. It clearly identifies the peculiar morphology of carbon fibers and the small particles of this material after HEBM. 

**Figure 7:**
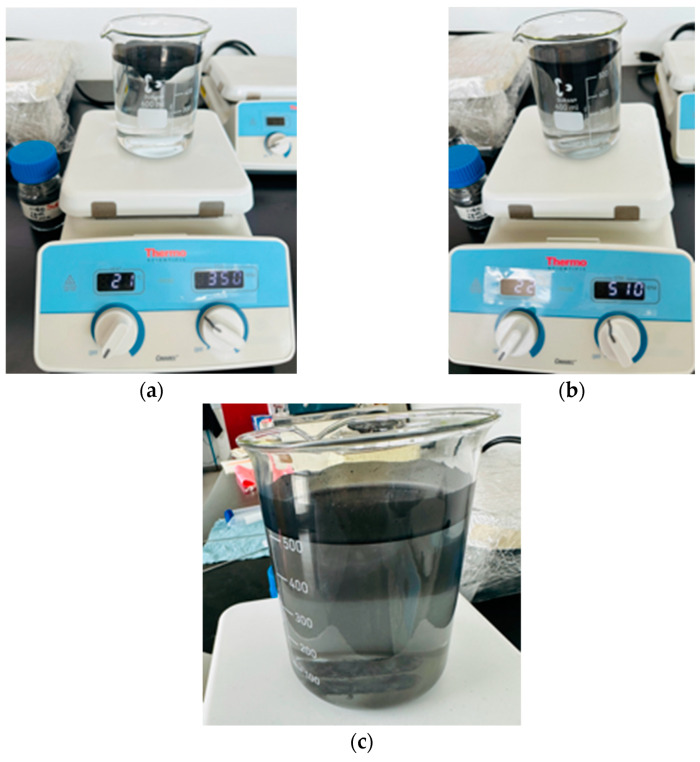
Photographs corresponding to the magnetic stirring process: (**a**) 350 rpm, (**b**) 510 rpm, (**c**) after stirring.

Continuing with the effort to separate the components of the recyclates (carbon fiber, epoxy resin, and rCFRP particles), a magnetic stirring test followed by ultrasound was carried out using the recyclates HEBM. Magnetic stirring was performed at 350 rpm for 10 min and 510 rpm for another 10 min (Figure 7a,b, respectively). During the test, the formation of a vortex, a product of magnetic stirring, was more pronounced as the stirring speed increased.

At the end of the test, and after 10 min, most of the HEBM recyclates remained on the surface, although a small amount of the sample precipitated, as shown in Figure 7c. According to the literature, carbon fiber and epoxy resin density is 1.75–2.00 g/cm^3^ and 1.2–1.3 g/cm^3^, respectively. In our case, the Epolam 2015 has a 1.15 g/ cm3 density. Therefore, the recyclates were expected to sink to the bottom of the beaker. 

During the magnetic stirring test, it was observed that the recyclates showed highly hydrophobic behavior, which could explain their ability to float in water.

**Figure 8:**
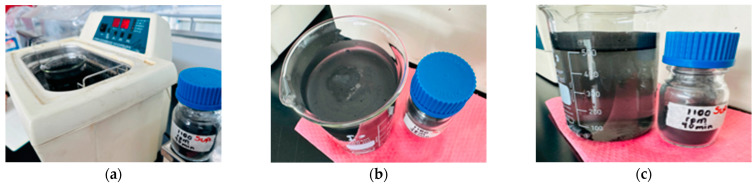
Photographs corresponding to the (**a**) ultrasonic bath process, (**b**) floating recyclates, (**c**) precipitated HEBM recyclates after 30 min.

The same batch of HEBM recyclates was subjected to an ultrasonic bath for 40 min (Figure 8a) to separate the components of the recyclates through cavitation bubbles induced by high-frequency pressure waves.

At the end of the test, the recyclates remained on the water surface (Figure 8b). However, precipitates and some particles suspended in the water inside the beaker were observed, as seen in Figure 8c. 

**Figure 9:**
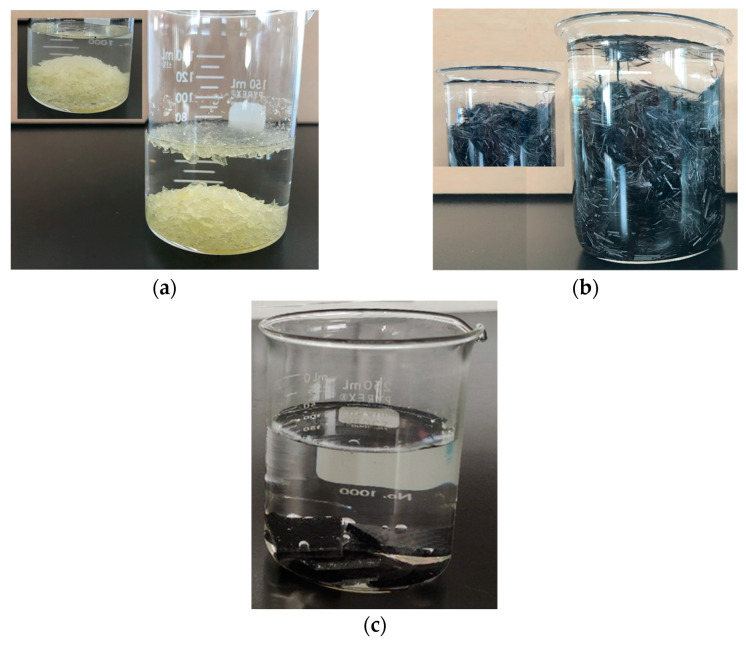
Photographs corresponding to (**a**) crushed epoxy resin, (**b**) short carbon fiber, and (**c**) CFRP samples submerged in distilled water.

To elucidate the flotability of HEBM recyclates, flotation tests of the recyclate constituents were performed in distilled water. First, the epoxy resin was evaluated. For this purpose, a 5 cm × 5 cm × 1 cm epoxy resin plate (Epolam 2015) was prepared. The resin plate was wrapped in a cloth and manually crushed into small pieces using a hammer. Approximately 10 g of ground epoxy resin was placed in the beaker, as shown in Figure 9a.

Most resin initially sinks, although some resin remaining floats. When stirring with a glass rod, most of the epoxy resin sinks to the bottom, and only a tiny proportion floats. A closer look (image box in Figure 9a) shows that some resin chunks floating in water are tiny and appear to have internal bubbles induced during the epoxy board manufacturing process. These bubbles and the aspect ratio of the particles could promote floating in water. Subsequently, the same test was performed using short carbon fiber (5–10 mm), as presented in Figure 9b. The carbon fiber was cut from the plain weave fabric used in this work. Approximately 10 g of short carbon fiber was placed in the beaker. The behavior was similar to that observed with the epoxy resin. A significant proportion of the short carbon fiber sinks, but some short carbon fibers float because of surface tension, so when pushing the fiber with laboratory tweezers or stirring it with a glass rod, the surface tension breaks, and the fiber sinks to the bottom of the beaker (image box in Figure 9b).

The epoxy particles and the short carbon fibers float because of the high aspect ratio of the particles. Edward Bormashenko [37] published that capillary forces better support more elongated bodies of a fixed volume because of an increase in the perimeter of the triple line, which is what occurs in the flotation of slender bodies, similar to what has been observed with particles (flakes) of epoxy resin and carbon fibers.

It is important to note that when the resin particles and carbon fiber were removed from the beaker, both were completely soaked in water. 

On the other hand, some pieces of CFRP composites were cut and immersed in distilled water, as shown in Figure 9c. As expected, the carbon fiber composite pieces sank immediately. However, several bubbles were observed to form. The bubbles could indicate that the composites have imperfections (small holes) that contain air.

**Figure 10:**
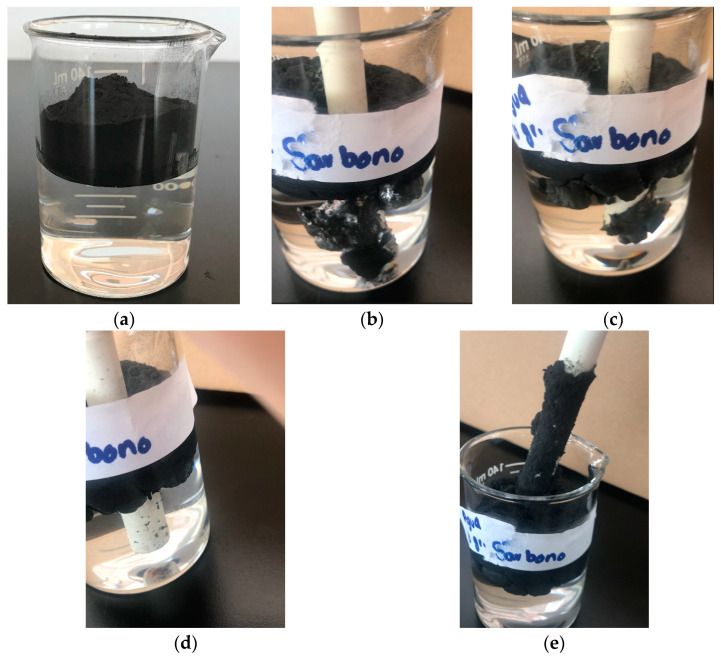
Photographs corresponding to (**a**) recyclates floating in distilled water, (**b**) recyclates adhering to PVC road submerged in distilled water, (**c**) recyclates detached from PVC road after stirring, (**d**) completely detached recyclates that return to the surface, and (**e**) recyclates adhered to PVC road outside the beaker.

Figure 10 shows the HEBM recyclate (powder) floating in distilled water. Contrary to what might be expected, the recyclate floats noticeably. The recyclate was stirred with a glass rod and remained afloat. Manual shaking was also carried out, observing that the recyclate did not become wet and remained floating. Only small particles sink after manual shaking. Finally, a PVC plastic rod was introduced to the beaker, showing how the recyclate adheres to the PVC rod, as shown in Figure 10b. The recyclate remains adhered even after stirring the PVC rod a little while immersed in water. Continuing with the stirring, some agglomerated pieces of recyclate powder detach from the rod and return to the surface, as seen in Figure 10c. A more vigorous stirring allows all the adhered and agglomerated recyclate to detach from the PVC rod and return to the surface (Figure 10d). However, a considerable proportion of recyclate adheres to its surface when the PVC rod is removed, as presented in Figure 10e. It is relevant to consider the appearance of shiny reflective surfaces (Figure 10b), which could be attributable to the occurrence of water–air interfaces.

The recyclate seems to have an electrostatic charge induced during the HEBM process, which promotes the agglomeration of the recyclate. If we also consider what is observed in Figure 2, where the particles of the composites seem to melt because of the friction of the end-mill cutting process, then we can suggest the particles contain trapped air, which promotes the floating of the recyclate.

Based on the above results, the floating of the recyclate would be due to the combination of three situations: surface tension, trapped air, and the electrostatics induced by the processing [38].

It seems that the induced electrostatics would be influencing the hydrophobicity of the recyclate. Mateusz Kruszelnicki et al. [39] indicated that flotation can be derived from the wettability of particles. The results showed that for the flotation process to occur, the minimum (critical) value of the contact angle must be exceeded, which was determined to be approximately 25° when the electrostatic interactions were attractive or weakly repulsive and up to 62° for strongly repulsive electrostatic interactions.

Other authors [40] experimented with floating metals, taking advantage of the energy of the water surface. The authors attribute this effect to the enhanced flotation ability of nanostructures on the surfaces of copper and stainless steel sheets. Sufficiently thin hydrophobic metals can slowly float underwater by trapping air on the surface.

It is well known that the ability of materials to sink or float depends on their density, shape, and the amount of water they displace [41,42]. Guo et al. [43] mentioned that this ability is further enhanced by superhydrophobic surfaces that exhibit water-repellent behavior as it provides a layer of air to replace volumes of water for increased buoyancy. Furthermore, attached air bubbles or layers can enhance flotation ability [44]. Edward Bormashenko [37] studied the flotation of bodies with different geometries, showing that more elongated bodies float thanks to capillary forces, taking into account the effect of the aspect ratio of the bodies. Thus, when the lateral dimension of the floating bodies is much smaller than the length of the capillary, the buoyancy is negligible, and the flotation is determined by the surface tension, according to what was studied by Dominic Vella [45].

**Figure 11:**
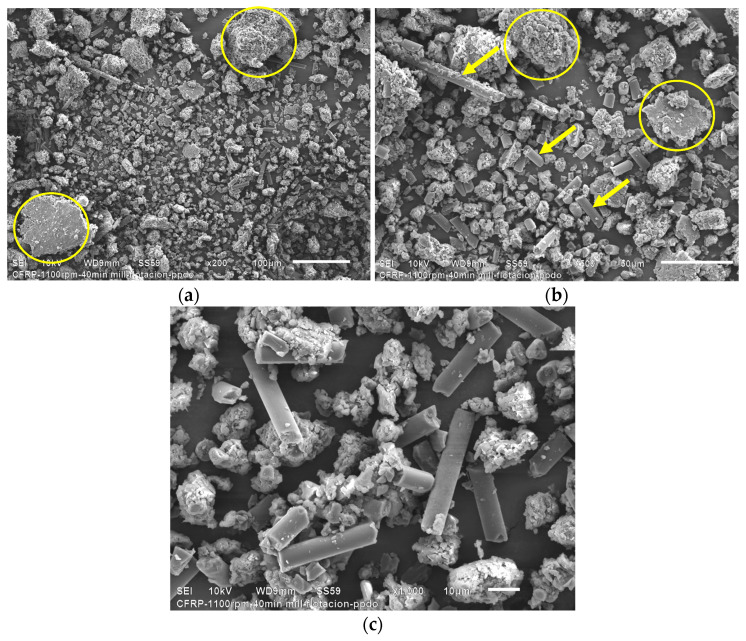
SEM micrographs corresponding to the precipitated HEBM recyclates after ultrasonic bath. Features of recyclates can be observed at different magnifications: (**a**) 200×, (**b**) 500×, (**c**) 1000×. The yellow circles highlight agglomerated particles, and the yellow arrows indicate single carbon fibers and graphite particles.

Figure 11 presents the SEM micrographs corresponding to the morphological analysis of the precipitated HEBM recyclates.

When comparing the micrographs corresponding to the HEBM recyclates with the HEBM ultrasonic recyclates at high magnifications (Figure 4c and Figure 11c), the HEBM ultrasonic recyclates contain a more significant amount of epoxy resin particles than the HEBM recyclates. The above would indicate that the precipitates are mostly epoxy resin, which allows progress toward a process of separation of the components of the recyclates [35]. In both cases, carbon fibers with some epoxy resin can be seen, which would indicate that the ultrasonic does not produce significant changes in the morphology of the recyclates, referring to the dispersion or separation of the epoxy resin adhered to the surface of the carbon fiber.

Resin particles are agglomerates that may contain graphite particles and short carbon fibers because of the strong molecular interaction between both constituents. The interface between the carbon fiber and the resin matrix plays a fundamental role in controlling the overall properties of the composites. Polyacrylonitrile (PAN) is the most common precursor for manufacturing carbon fibers, so the mechanical and electrochemical properties of the fibers largely depend on the nature of the preparation methods and subsequent processing treatments. The modification of carbon fibers is expected to improve their structural performance. Thus, their reinforcing performance often depends on the interfacial bonding strength between the fibers and the resin matrix. Unmodified carbon fibers compromise the mechanical properties of the composite [46]. 

Ultrasonication is the technique adopted to reduce the size of clay particles and disperse carbon nanotubes [47]. Ultrasonication produces cavitation bubbles that collapse rapidly, leading to impact shock waves and interparticle collisions. In this way, sonication induces the disaggregation of particles and favors their dispersion depending on the ultrasonication energy [48]. 

**Figure 12:**
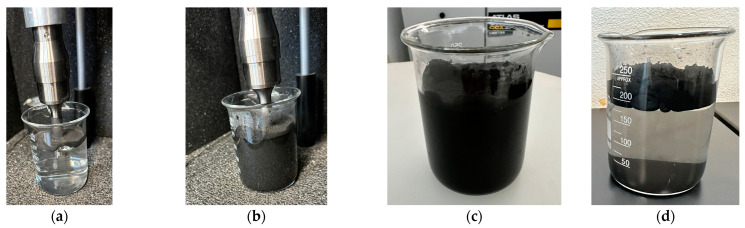
Photographs corresponding to the ultrasonication process: (**a**) sample 1 g, (**b**) sample 2 g, (**c**) sample 2 g after sonication, (**d**) sample 2 g after 30 min that reveals the separation of floated and precipitated recyclates.

Ultrasonication was carried out using samples of HEBM recyclates and sieved end mill recyclates (<90 microns). Figure 12 presents the sequence of the experimental ultrasonication procedure performed on the HEBM recyclates and the sieved end mill recyclates. Four experimental ultrasonication tests were run that consisted of varying the amplitude (50–75%) and the content of recyclates (1–2 g). The temperature was monitored during the experimental tests. For an amplitude of 50%, the test exceeded 60 °C after 15 min of ultrasonication, while for an amplitude of 75%, the test exceeded 60 °C after 10 min of operation. Observations in the recipient of water/powder solution were that it evaporated and spread the material that floats on the surface. This solution behavior could not be controlled during ultrasonication, and cooling was performed by placing the recipient in a vessel with cold water, which required approximately 10 or 15 min, depending on the energy sonication used to reach 25–27 °C in the solution.

Figure 12a corresponds to the 1 g sample of HEBM recyclates, while Figure 12b corresponds to the 2 g sample of HEBM recyclates. The physical difference in using 1 or 2 g of recyclates is seen. Using 2 g of recyclates subjected to an amplitude of 75% produced a considerable amount of bubbles, which could promote the effective separation of the components of the recyclates. After ultrasonication, the suspension remains completely black (Figure 12c). After 30 min, the suspension cools, and the floating recyclates and precipitates are separated, as shown in Figure 12d. As the content of recyclates increases, the precipitates increase. A similar behavior was observed in the sieved end mill recyclates. Following the methodological sequence of the research, the morphology of the precipitated recyclates was evaluated by SEM.

**Figure 13:**
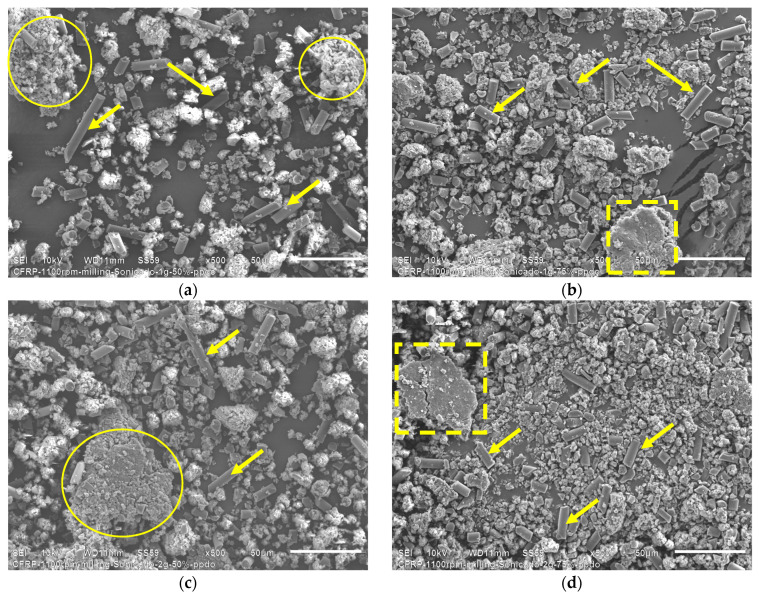
SEM micrographs corresponding to the ultrasonicated precipitates of HEBM recyclates: (**a**) 1 g and 50% amplitude, (**b**) 1 g and 75% amplitude, (**c**) 2 g and 50% amplitude, (**d**) 2 g and 75% amplitude. The yellow circles highlight agglomerated particles, and the yellow arrows indicate single carbon fibers and graphite particles. The yellow dashed square indicates fractured particles.

**Figure 14:**
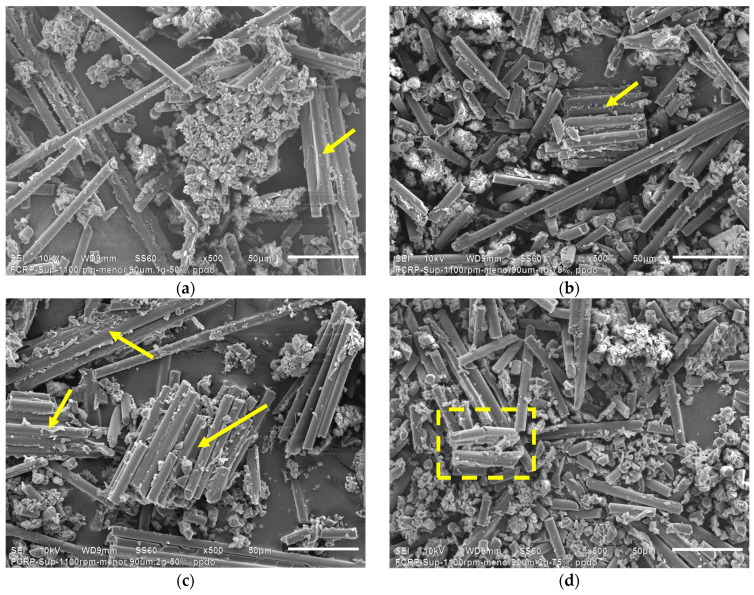
SEM micrographs corresponding to the ultrasonicated precipitates of sieved end mill recyclates: (**a**) 1 g and 50% amplitude, (**b**) 1 g and 75% amplitude, (**c**) 2 g and 50% amplitude, (**d**) 2 g and 75% amplitude. The yellow arrows indicate rCFRP particles and the yellow dashed square indicates fractured carbon fiber.

Figure 13 presents the SEM micrographs corresponding to the ultrasonicated precipitates of HEBM recyclates, while Figure 14 presents the SEM micrographs of the ultrasonicated precipitates of sieved end mill recyclates. 

When comparing the SEM micrographs of the ultrasonicated HEBM recyclates, it does not seem—at first glance—that there are evident changes in the morphology of the precipitates. However, significant morphological variations can be observed if we compare the morphologies of the recyclates subjected to a lower amplitude and low residue content (Figure 13a) with the tests carried out with recyclates of 2 g and higher amplitude (Figure 13d). Ultrasonication breaks down the hard epoxy resin particles and reduces their size. Cracks are observed in some larger epoxy particles, as highlighted in a dashed square indicated with arrows in Figure 13. With 2 g of recyclates and 75% amplitude, a greater content of graphite particles and a considerable amount of epoxy resin microparticles can be observed, much smaller than those observed in Figure 13a.

It is possible to appreciate that the morphology of the precipitates corresponding to the sieved and ultrasonicated end mill recyclates differs from the HEBM recyclates. The first difference is that individual long carbon fibers, agglomerated CFRP particles, and some epoxy resin particles are observed. Most of the epoxy resin is attached to the carbon fibers. Contrary to what is observed in Figure 2c, the particles present carbon fibers aligned and homogeneously distributed (Figure 14b,c). Figure 14d, which represents the morphology of 2 g recyclates subjected to high amplitude ultrasonication, shows individual carbon fibers and dispersed epoxy resin particles. Ultrasonication separates the carbon fibers and releases the epoxy resin from their surface, so a higher content of micrometric epoxy resin particles distributed between the individual carbon fibers is observed. In addition, the carbon fibers are less long because ultrasonication breaks the agglomerates and fragments the fibers, as seen in Figure 14d. The results confirm that ultrasonication is effective in sifted end mill recyclates since the carbon fibers are released from the epoxy resin and break into tiny agglomerated particles.

The strategies evaluated in the mechanical recycling of CFRP composites allow us to assess the differences between end mills and HEBM recycling. HEBM recycling requires small samples to enter the ball mill; a prior cutting or grinding step is necessary. HEBM recycling allows us to obtain small particles of recyclates, but it is impossible to separate their components: carbon fiber, epoxy resin, and CFRP particles.

Mill recyclates are obtained directly by cutting large CFRP parts or components. The recyclates obtained are a combination of long fibers and micrometric particles. A sieving step allows for particle homogeneity. However, large carbon fibers can pass the sieve.

Ultrasonication does not significantly affect HEBM recyclates because of the high energy they are subjected to during the grinding process, but it is effective on end-mill recyclates. The ultrasonication amplitude has a greater impact than the content of the samples in terms of the ability to separate the epoxy resin from the carbon fiber.

## 4. Conclusions

The morphology of the recyclates obtained through different mechanical recycling techniques and subsequent treatments was analyzed to separate the constituents of the recyclates, which are carbon fiber, epoxy powder, and CRFP particles.

The sieving of the recycle end mill separates the waste, although individual carbon fibers manage to pass through the wire cloth.

The ultrasonic bath did not present a significant difference in separating the epoxy resin from the carbon fiber.

The end mill produces CFRP particles, epoxy resin agglomerated waste, and some individual fibers with epoxy resin on their surface.

The HEBM process produces tiny particles of graphite and epoxy resin.

Ultrasonication separates CFRP into individual long carbon fibers, breaks down the epoxy resin particles to make them smaller, fragments the carbon fibers, and separates the epoxy resin from the surface of the fibers.

Ultrasonication allows effective separation of the constituents from the recyclates. However, the procedure is time-consuming and at a laboratory level. At the moment, it is impossible to collect the recyclates’ constituents separately.

CFRP constituents do not float in distilled water or tap water. However, the recyclates float. The end mill and HEBM waste production process promote the presence of trapped air and electrostatics, which allows recyclates to float in water and be hydrophobic. The appearance of shiny reflective surfaces could be attributable to the occurrence of water–air interfaces.

The results of this research present progress towards developing technology for the mechanical recycling of CFRP and open a range of opportunities in reusing recyclates to create new products.

## Figures and Tables

**Table 1:** Chemical composition of recyclates.

Process	End Milling	HEBM
Element	Concentration (Wt.%)	Concentration (wt.%)
Cr	0.0023	0.213
Fe	0.0123	1.566
Co	0.0017	0.132

## Data Availability

The original contributions presented in the study are included in the article/Supplementary Materials, further inquiries can be directed to the corresponding author.

## Supplementary Materials

The following supporting information can be downloaded at: https://www.mdpi.com/article/10.3390/polym16162350/s1, Figure S1: Particle size evaluation: (a) Carbon fiber, (b) epoxy resin particles. Figure S2: Several SEM microgprahs taken from different zones. Figure S3: Punctual analyses where we observed peaks of carbon and oxygen. Figure S4: Process of ultrasonication. Figure S5: Photographs corresponding to (a) crushed epoxy resin, (b) 10 mg of chopped carbon fiber, (c) 5 mg of chopped carbon fiber submerged in tap water. Figure S6: Photograph corresponding to the resin chunks that float in tap water. Video S1: Adherence rCFRP by electrostatic.
