# Supplementary material for: Technologies for Mechanical Recycling of Carbon Fiber-Reinforced Polymers (CFRP) Composites: End Mill, High-Energy Ball Milling, and Ultrasonication
Source: Polymers (Basel). 2024 Aug 20;16(16):2350. doi: 10.3390/polym16162350 (PMC11359413; doi:10.3390/polym16162350)
Supplement: Supplementary file 1 [file polymers-16-02350-s001.zip › polymers-3047891-supplementary.pdf]

## Complementary Information

Particle size evaluation of powder obtained by HEBM was performed by using the ImageJ free-software using 5 SEM-images and it has been considered two types of particles: C-fiber and epoxy. Results are shown in following Figure.

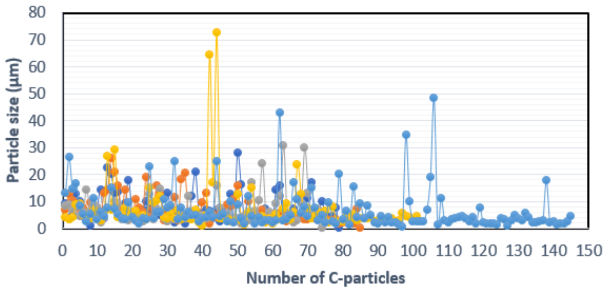


(**a**)

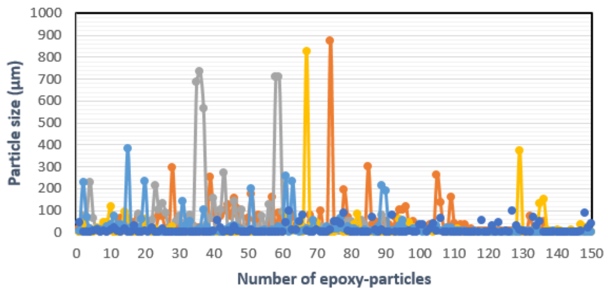


(**b**)

Figure S1: Particle size evaluation: (a) Carbon fiber, (b) epoxy resin particles.

It is observed the large difference in particle size comparing the C-fibers, some up to 80 microns vs. epoxy which have particles near to 900 microns. However, the average size of particles are 7.06 and 32.86 microns of C-fibers and epoxy, respectively. The average of C-fibers is considered as in range of submicrometric size.

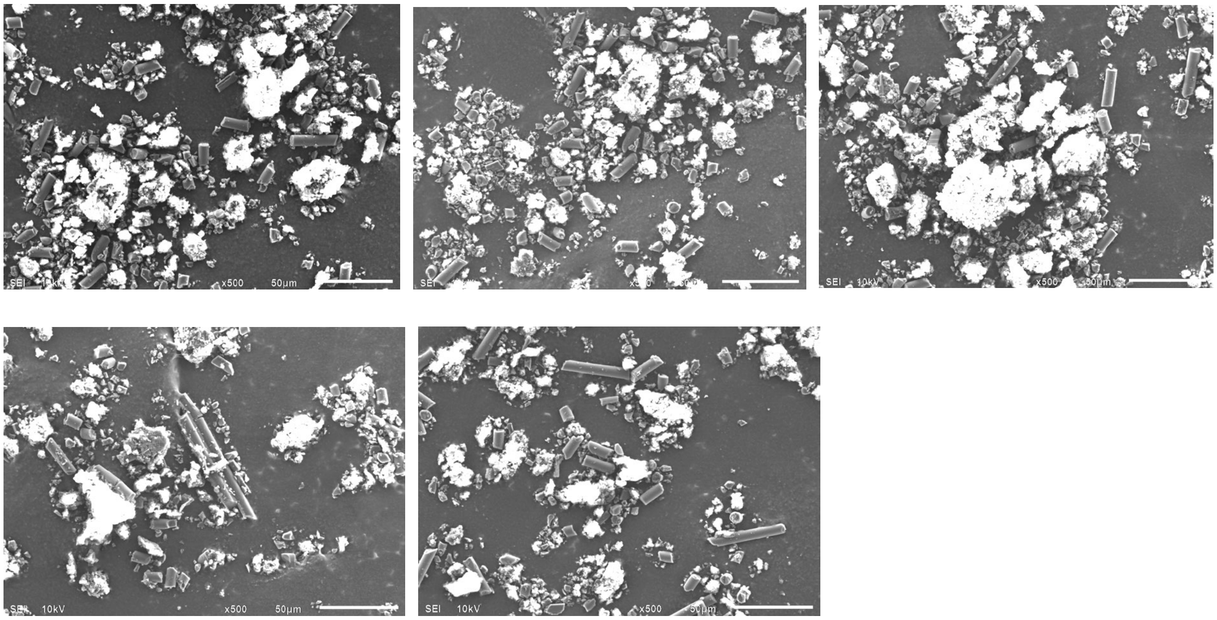

Figure S2: Several SEM microgprahs taken from different zones.

Different punctual analyses where we observed peaks of carbon and oxygen, as presented in Figure 5 of the manuscript. However, we add different results here.

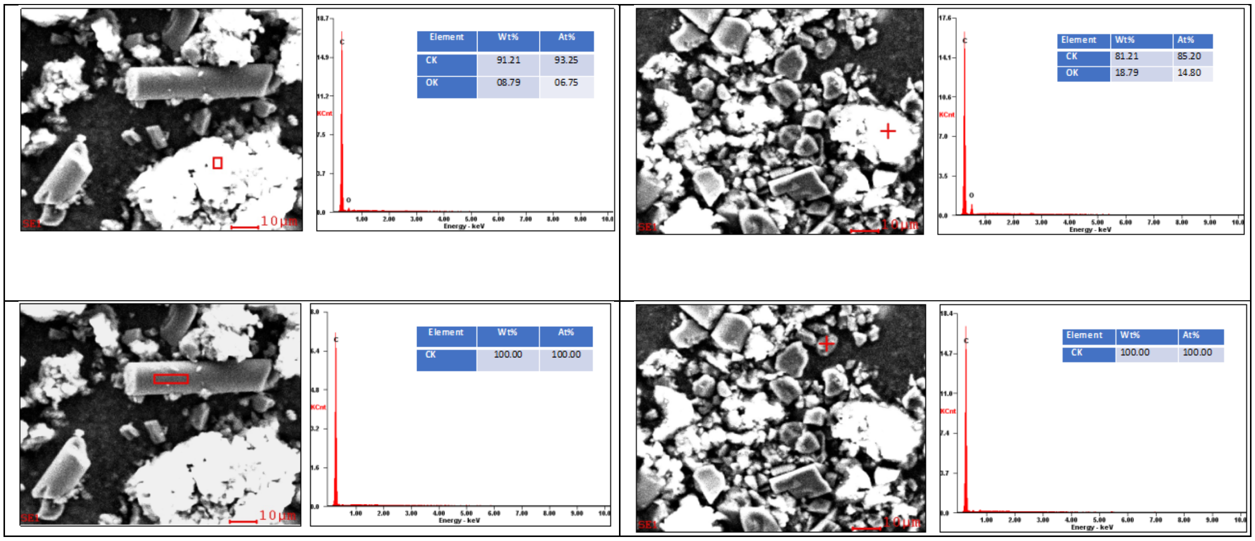


Figure S3: Punctual analyses where we observed peaks of carbon and oxygen.

Observations in the recipient of water/powder solution were that it started to evaporate and spread the material that floats on the surface. This solution behavior could not be controlled during ultrasonication, and cooling was performed by placing the recipient in a vessel with cold water, which required 10 or 15 minutes approximately, depending on the energy sonication used to reach 25-27 °C in the solution.

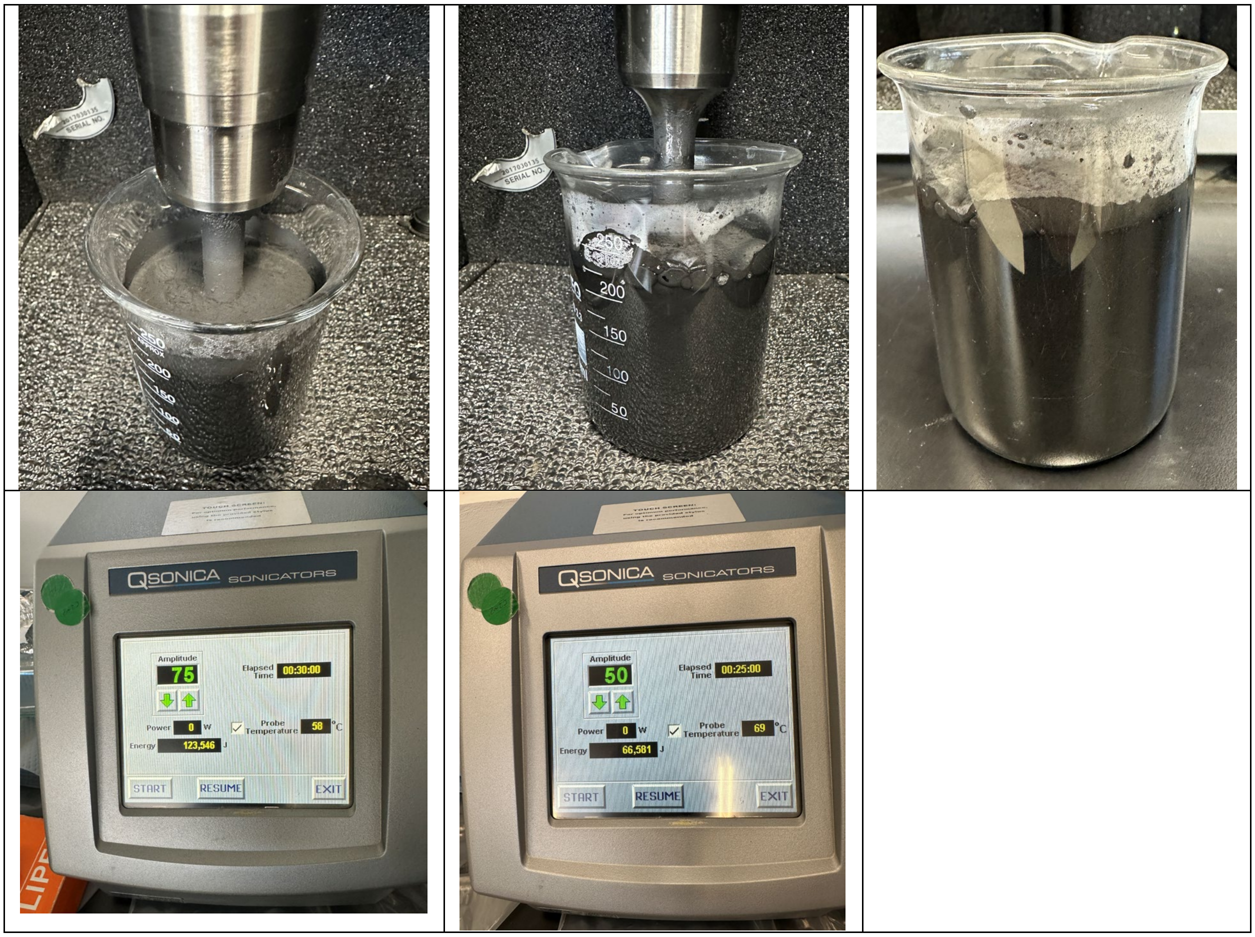


Figure S4: Process of ultrasonication.

To elucidate the flotability of HEBM recyclates, flotation tests of the recyclate constituents were also performed in tap water. The following Figure reveals that epoxy resin and short carbon fibers show similar behaviors. Some parts sinks, and a small part floats. Additionally, 10 g and a 5 g sample were placed in the tap water beakers.

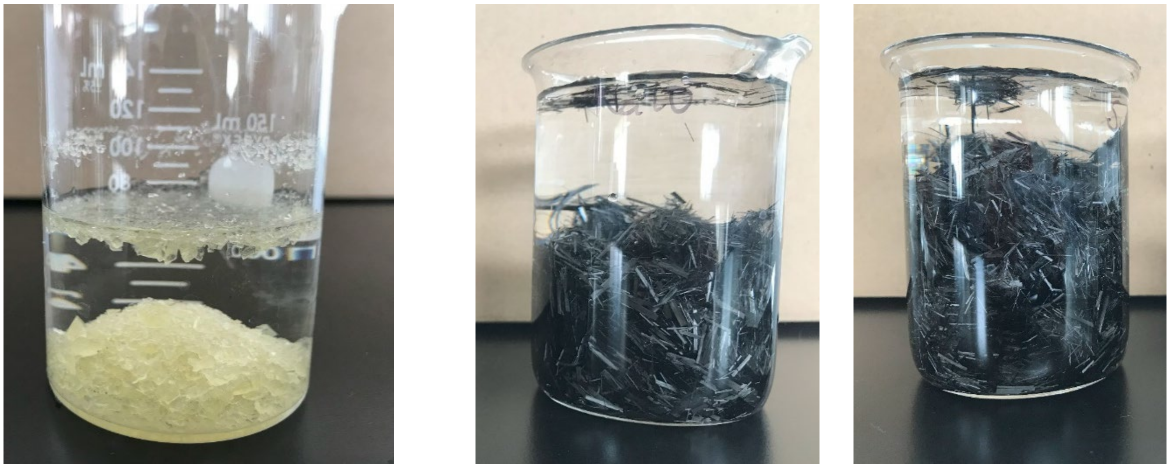

a) b) c)

Figure S5. Photographs corresponding to a) crushed epoxy resin, b) 10 mg of chopped carbon fiber, c) 5 mg of chopped carbon fiber submerged in tap water.

The chopped fibers can float due to the high aspect ratio of the particles and the surface tension, so when pushing the fiber with laboratory tweezers or stirring it with a glass rod, the surface tension breaks and the fibers sink to the bottom of the beaker.

Epoxy resin can float due to the high aspect ratio of the particles. After stirring it with a glass rod, most epoxy sinks to the bottom of the beaker. The following Figure reveals that the resin chunks that float in water are tiny and appear to have internal bubbles induced during the epoxy board manufacturing process. These bubbles and the aspect ratio of the particles could promote floating in water.

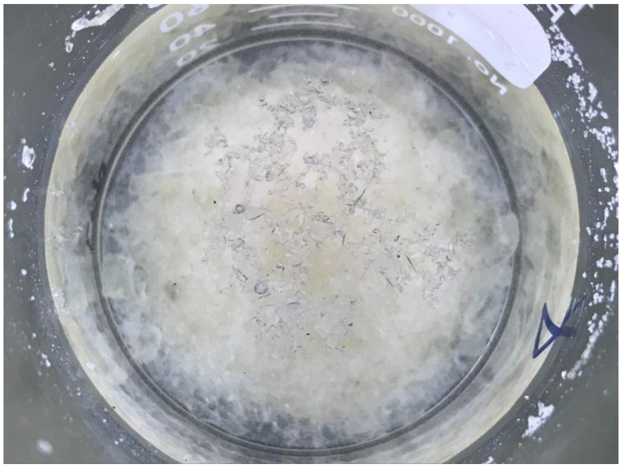

Figure S6. Photograph corresponding to the resin chunks that float in tap water.

It was clearly observed how all the materials sink, whether in tap or distilled water. Therefore, the type of water does not influence the flotation of the carbon fiber or the epoxy resin. The material content (5 or 10 g) does not seem to influence either.
